# Assessment of Clinical Guideline Use in the Prevention, Diagnosis, and Treatment of Cryptococcal Meningitis Among Health Care Providers in Ethiopia

**DOI:** 10.1093/ofid/ofaf715

**Published:** 2025-11-24

**Authors:** Blen M Gebresilassie, Gerome B Vallejos, Ryan S Walker, Omosefe Noruwa, Kathryn B Holroyd, Temesgen Nurye, Carla Kim, Biniyam Ayele, Kiran T Thakur

**Affiliations:** Amanuel Mental Specialized Hospital, Addis Ababa, Ethiopia; Program in Neuroinfectious Diseases, Department of Neurology, Columbia University Irving Medical Center, New York Presbyterian Hospital, New York, New York, USA; Program in Neuroinfectious Diseases, Department of Neurology, Columbia University Irving Medical Center, New York Presbyterian Hospital, New York, New York, USA; Program in Neuroinfectious Diseases, Department of Neurology, Columbia University Irving Medical Center, New York Presbyterian Hospital, New York, New York, USA; Harvard University, Cambridge, Massachusetts, USA; Program in Neuroinfectious Diseases, Department of Neurology, Columbia University Irving Medical Center, New York Presbyterian Hospital, New York, New York, USA; Program in Neuroinfectious Diseases, Department of Neurology, Columbia University Irving Medical Center, New York Presbyterian Hospital, New York, New York, USA; Global Brain Health Institute (GBHI), University of California, San Francisco (UCSF), San Francisco, California, USA; Program in Neuroinfectious Diseases, Department of Neurology, Columbia University Irving Medical Center, New York Presbyterian Hospital, New York, New York, USA; Global Brain Health Institute (GBHI), University of California, San Francisco (UCSF), San Francisco, California, USA; Program in Neuroinfectious Diseases, Department of Neurology, Columbia University Irving Medical Center, New York Presbyterian Hospital, New York, New York, USA

**Keywords:** clinical guidelines, cryptococcal meningitis, Ethiopia, health care practices, HIV

## Abstract

**Background:**

Cryptococcal meningitis (CM) is a significant cause of morbidity and mortality in individuals with advanced HIV. Ethiopia, with a high HIV burden and significant socioeconomic challenges, has limited data on CM. Understanding clinical practices is crucial to identifying gaps and improving disease management.

**Methods:**

A survey was conducted among health care workers (HCWs), microbiologists, and pharmacists across 9 Ethiopian health facilities from July to November 2024. Eligibility required active involvement in HIV or CM care. Ethics approval and informed consent were secured. Three profession-specific questionnaires were used to assess knowledge of CM care and challenges. Data were analyzed in R, version 4.3.2.

**Results:**

A total of 311 participants were included: HCWs, n = 202; microbiologists, n = 65; and pharmacists, n = 44. Most participants, 188/202 (93.1%) HCWs, 42/44 (95.5%) pharmacists, and 54/65 (83.1%) microbiologists, recognized the need for additional guideline training. Only 59/202 (29%) HCWs reported that screening for cryptococcal antigenemia was always performed. Among antifungals, fluconazole was the most available, with 21/44 (48%) pharmacists and 49/202 (24%) HCWs reporting it as always in stock. Liposomal amphotericin B (L-Amb) was reported as available by only 7/44 (16%) pharmacists and 44/202 (22%) HCWs. The most significant barriers to guideline implementation were insufficient policy support and challenges in integrating guidelines into electronic health records, as reported by 152/202 (75.2%) and 143/202 (70.8%) HCWs, respectively.

**Conclusions:**

There are substantial gaps in CM care, highlighting the importance of improving resource allocation, technical assistance, additional training, and financial support.

## Teaser

This study identifies major gaps in the prevention, diagnosis, and treatment of Cryptococcal meningitis in Ethiopia. Findings highlight limited training, poor diagnostic access, and treatment barriers, emphasizing the need for improved guideline implementation, resources, and support for healthcare providers.

Cryptococcal meningitis (CM) remains one of the most significant life-threatening opportunistic infections among people with HIV (PWH), especially in regions with high prevalence of advanced HIV and limited health care resources [1]. Globally, CM accounts for 19% of AIDS-related mortality, with Africa bearing ∼75% of this burden. Delayed diagnosis and treatment significantly increase the morbidity and mortality of CM [1, 2].

In Ethiopia, where HIV prevalence is high, studies have found that blood cryptococcal antigen (CrAg) positivity is 20.9% among individuals with CD4 counts <150 cells/mm³ and 11.4% among those with CD4 counts ≤100 cells/mm³ at major hospitals [3, 4]. CrAg can be detected in the bloodstream before the onset of clinical symptoms, offering a crucial window for early treatment and intervention to halt disease progression [5–7]. However, despite this opportunity, barriers such as limited access to screening tests, clinical assessment, and timely lumbar puncture (LP) persist, often leading to late-stage diagnoses and significantly increasing mortality risk.

Additionally, even in cases where diagnosis is possible, resource limitations and treatment inconsistencies still result in unacceptably high mortality [2, 8–11]. In 2022, the World Health Organization (WHO) released updated CM guidelines designed to streamline care and reduce CM-related mortality by emphasizing early detection and standardized induction therapy [9]. However, overall implementation remains difficult, as evidenced by low adherence to CM guidelines among health workers in Uganda [10]. Although Ethiopia has adopted the 2022 WHO guidelines into national health care policy, the extent to which they are implemented in clinical practice remains largely unknown. Identifying real-world barriers, such as medication shortages, diagnostic limitations, and training deficiencies, is the first step toward effective intervention [11]. This study therefore aims to characterize current practices of and identify challenges for health care professionals caring for people with CM. Additionally, this study evaluates the adoption of CM guidelines to provide actionable recommendations for improving patient outcomes.

## METHODS

### Study Design

**Figure 1.**
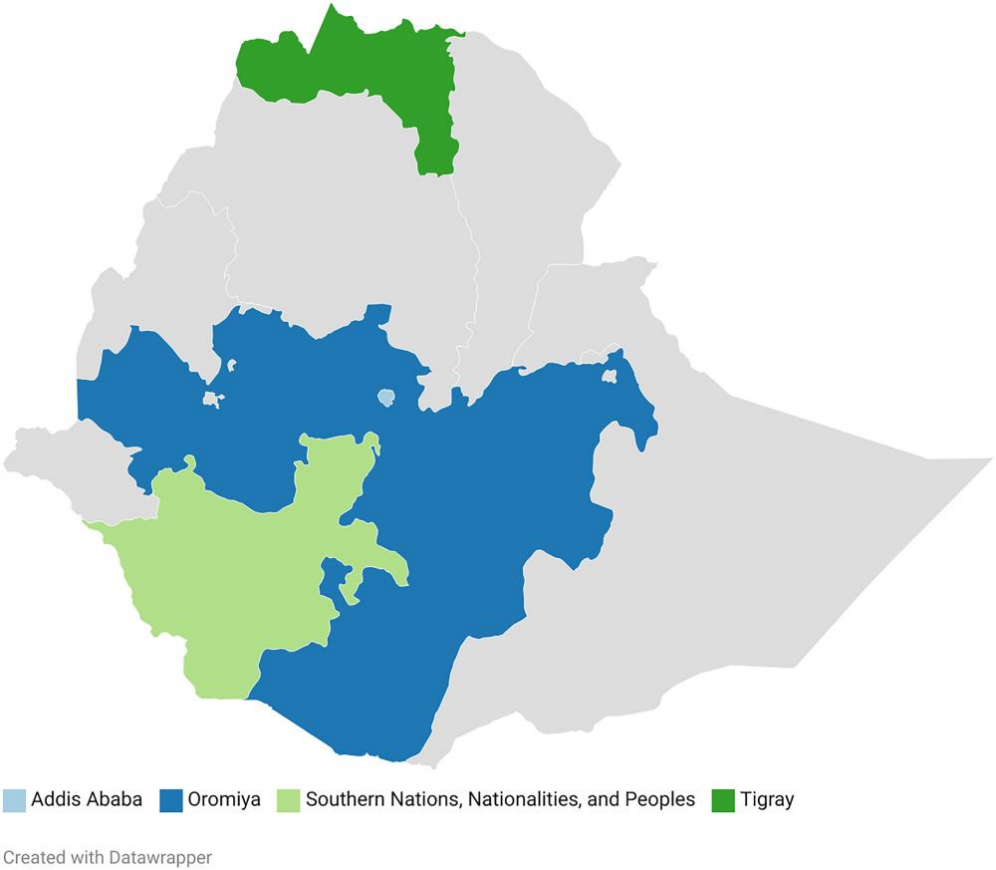
Geographic distribution of survey locations across Ethiopian regions.

This observational study surveyed health care professionals including health care workers (HCWs: physicians, nurses, and health officers), microbiologists, and pharmacists across 9 health facilities, including 4 academic institutions, 2 referral hospitals, 2 health centers, and 1 private specialized neurology center located in Addis Ababa. Physicians act as primary decision-makers, responsible for patient evaluation, ordering diagnostic tests, formulating treatment plans, and coordinating follow-up care. Nurses’ roles vary by setting but generally include HIV testing, assessing opportunistic infections, ordering CD4 and viral load tests, managing medication refills, monitoring side effects, and collaborating with physicians; in some remote primary care settings, nurses may also provide physician-level care. Microbiologists perform laboratory diagnostics such as CD4 counts, viral loads, chemistry panels, and cultures. Pharmacists, encompassing both dispensary and store roles, maintain comprehensive knowledge of medication availability, commonly prescribed drugs, their indications, and side effects through rotational assignments. To ensure regional diversity and representation of different care levels, the study included facilities from various parts of Ethiopia (Figure 1), encompassing academic institutions, referral hospitals, and health centers, all with dedicated HIV units. The facilities were selected based on a location free from active conflict, where internet was available and data could be collected. In addition, all facilities other than the health centers had a consultant neurologist to coordinate the survey. Data collection was performed between July and November 2024.

A purposive sampling technique was used to maximize the number of participants in each facility. The number of participants was proportionally allocated based on the population served and the number of health care workers at each facility. Eligibility criteria required that participants be actively involved in settings where HIV and/or cryptococcal meningitis cases might be encountered. The sole exclusion criterion was incomplete questionnaire responses. Ethics approval was obtained from the institutional review board (IRB) and ethics committee of all participating facilities. Informed consent was obtained from all participants before their inclusion in the study.

### Survey Design

Three questionnaires (HCW, microbiologist, and pharmacist) were designed to assess participants’ knowledge of the 2022 WHO CM guidelines, availability of diagnostic tools, standard treatment protocols, and reported challenges following CM guidelines. The final HCW survey was comprised of 2 sections: a demographic section and a knowledge section (Supplementary Data). The demographic section assessed respondents’ specific role, number of cases encountered, years of experience, practice location, guideline awareness, and training. The knowledge section evaluated key domains, including prevention and screening, diagnosis, resource availability, pharmacologic and adjunct interventions, follow-up, and barriers to care. A 5-point Likert scale (always, sometimes, occasionally, rarely, never) (Supplementary Data) was used to assess the frequency and confidence, while the others were open-ended questions. For microbiologists, the knowledge section included questions on diagnosis. Pharmacists answered questions on treatment.

### Statistical Analysis

Survey responses were imported into R, version 4.3.2, for data processing and analysis. Each response was recorded and systematically cleaned to ensure data integrity. Only fully completed surveys (100% response rate) were included in the final analysis to maintain data quality and avoid biases introduced by partial responses. Categorical variables were summarized using frequency distributions and expressed as counts (No.) and percentages (%). Stacked bar charts were used to illustrate the distribution for all variables across different groups, allowing for easy comparison of subgroup proportions.

All analyses and visualizations were conducted in R, version 4.3.3.

## RESULTS

A total of 311 participants were included in the study (Table 1), 202 HCWs, 65 microbiologists, and 44 pharmacists. Most respondents had 1–5 years of experience (106/202 [52.5%]), while few had >10 years (13/202 [6.4%]). Among microbiologists, 23/65 (35.4%) had 1–5 years of experience, while 10/65 (15.4%) had >10 years. Among pharmacists, 15/44 (34.2%) had 1–5 years of experience, while 10/44 (22.7%) had >10 years. In terms of clinical exposure to CM cases, 108/202 (53.5%) HCWs and 31/65 (47.7%) microbiologists reported having seen <5 CM cases, while 76/202 (37.6%) HCWs and 24/65 (36.9%) microbiologists reported encountering 5–20 CM cases. Lastly, only 18/202 (8.9%) HCWs and 10/65 (15.4%) microbiologists had seen >20 CM cases. The majority of HCWs (107/202 [53%]) and pharmacists (24/44 [54.6%]) were based at academic institutions, while microbiologists were more commonly employed in hospitals (23/65 [35.4%]). Awareness of the 2022 WHO CM guidelines was highest among microbiologists (51/65 [78.5%]) and lowest among pharmacists (15/44 [34.1%]). Only 11/202 (5.4%) HCWs had received training on CM guidelines, with microbiologists having the highest training rate (28/65 [43.1%]). Nearly all pharmacists (43/44 [97.7%]) had not received training. The majority (188/202 [93.1%]) of HCWs recognized the need for additional training, with particularly high demand among pharmacists (42/44 [95.5%]) and microbiologists (54/65 [83.1%]).

**Table 1.** Demographic Profile of Health Care Professionals

	Health Care Workers (n = 202)	Microbiologists (n = 65)	Pharmacists (n = 44)
Years of experience, No. (%)			
>10 y	13 (6.4)	10 (15.4)	10 (22.7)
6–10 y	27 (13.4)	13 (20)	8 (18.2)
1–5 y	106 (52.5)	23 (35.4)	15 (34.2)
<1 y	56 (27.7)	19 (29.2)	11 (25.0)
CM cases, No. (%)			
>20	18 (8.9)	10 (15.4)	
5–20	76 (37.6)	24 (36.9)	
<5	108 (53.5)	31 (47.7)	
Place, No. (%)			
Academic institution	107 (53)	16 (24.6)	24 (54.6)
Hospital	44 (21.8)	23 (35.4)	8 (18.2)
Health center	42 (20.8)	10 (15.4)	0 (0)
Other	9 (4.5)	9 (13.8)	12 (27.3)
Awareness of the 2022 WHO CM guidelines, No. (%)			
Yes	118 (58.4)	51 (78.5)	15 (34.1)
No	84 (41.6)	14 (21.5)	29 (65.9)
Received training on the 2022 WHO cryptococcal infection prevention, diagnosis, and treatment guidelines, No. (%)			
Yes	11 (5.4)	28 (43.1)	1 (2.3)
No	191 (94.6)	37 (56.9)	43 (97.7)
Do you believe there is a need for additional training?, No. (%)			
Yes	188 (93.1)	54 (83.1)	42 (95.5)
No	14 (6.9)	11 (6.9)	2 (4.5)

Abbreviations: CM, cryptococcal meningitis; WHO, World Health Organization.

### Prevention and Screening

**Figure 2.**
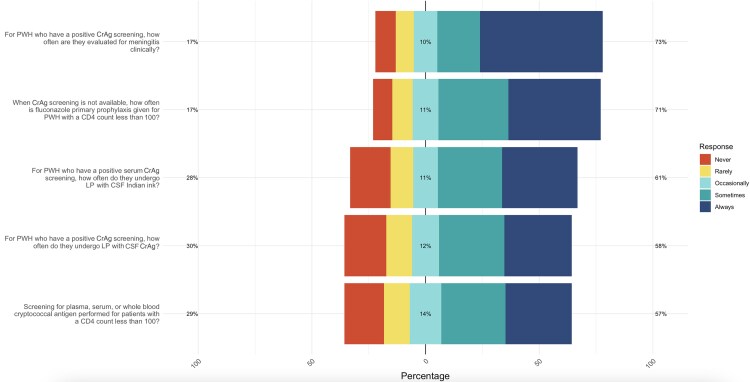
Prevention and screening practices according to HCWs. Abbreviations: CSF, cerebrospinal fluid; HCWs, health care workers; LP, lumbar puncture; PWH, people with HIV.

Screening for CrAg in patients with a CD4 count <100 cells/mm³ was reported as occasionally to always performed according to 144/202 (71%) HCWs, while 58/202 (29%) reported doing so rarely or never (Figure 2). Clinical evaluation for meningitis in PWH who tested positive for CrAg was more commonly implemented, with 168/202 (83%) occasionally to always performing it, while 34/202 (17%) reported rarely to never performing it. LP and India ink staining of cerebrospinal fluid (CSF) for PWH with a positive serum CrAg test were occasionally to always conducted by 168/202 (83%) HCWs, with another 34/202 (17%) reporting rarely to never performing this procedure. Similarly, LP and CrAg assay of CSF were conducted by 142/202 (70%) HCW, while 60/202 (30%) reported rarely to never implementing this measure. Fluconazole prophylaxis for PWH with a CD4 count <100 cells/mm³ when CrAg screening was unavailable was always given according to 167/202 (83%) HCWs, while 35/202 (17%) reported rarely to never implementing this measure.

### Diagnosis

#### Availability of Diagnostic Tests

**Figure 3.**
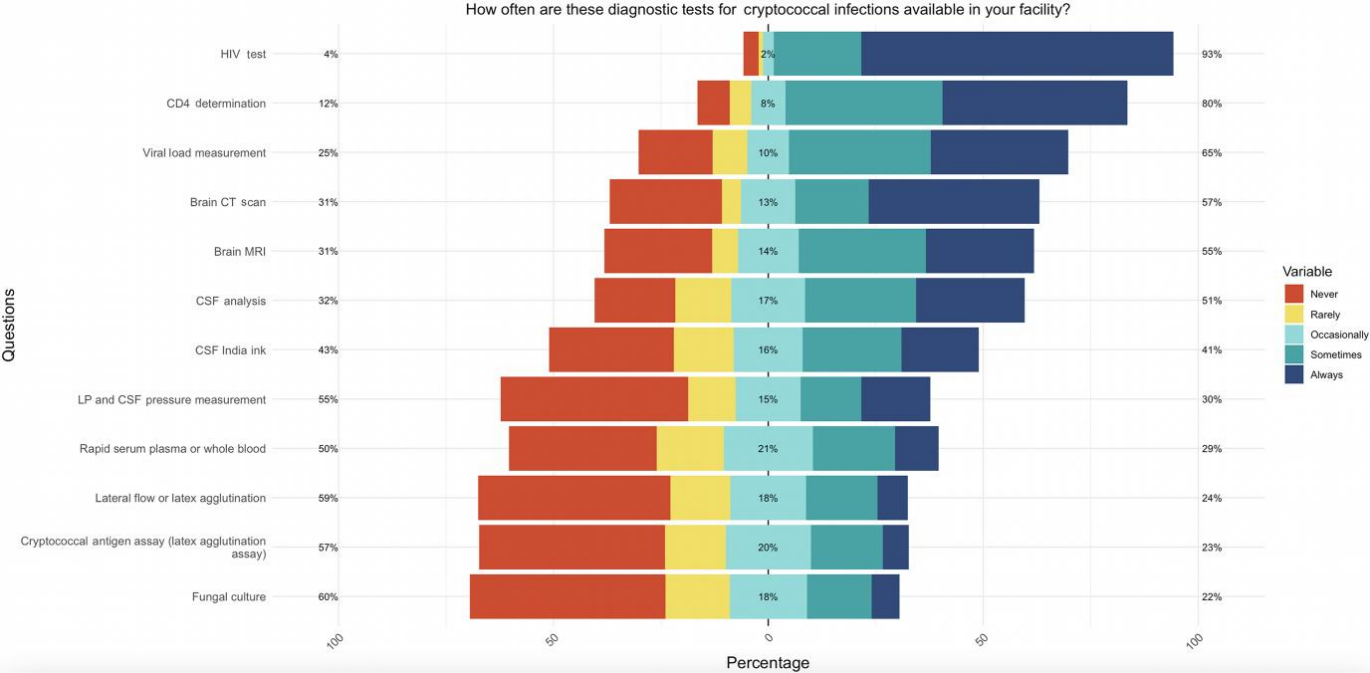
Diagnostic tests according to HCWs. Abbreviations: CSF, cerebrospinal fluid; CT, computed tomography; HCWs, health care workers; LP, lumbar puncture; MRI, magnetic resonance imaging.

Diagnostic test availability among HCWs varied widely (Figure 3). HIV testing was occasionally to always available according to 192/202 (95%) HCWs and 60/65 (92%) microbiologists. CD4 determination was occasionally to always available according to 175/202 (87%) and 61/65 (93%) HCWs and microbiologists, respectively (Supplementary Table 1). Viral load measurement was always available according to 147/202 (73%) HCWs, with 60/65 (92%) microbiologists occasionally to always performing it. Notably, CSF analysis was only available in 135/202 (25%), with 50/65 (76%) microbiologists occasionally to always using it. CrAg testing was occasionally to always available according to 99/202 (49%) HCWs and 50/65 (76%) microbiologists.

CrAg lateral flow and latex agglutination assays were rarely to never available, with 117/202 (58%) HCWs and 22/65 (34%) microbiologists. Fungal culture had the lowest availability and usage, with 121/202 (7%) HCWs and 24/65 (37%) microbiologists performing it rarely to never. Imaging tests like brain computed tomography and magnetic resonance imaging were also rarely to never available according to 61/202 (30%) and 62/202 (31%) HCWs, respectively.

#### Availability of Test Tools and Equipment

Cryptococcal infection guidelines were occasionally to always available according to 156/202 (77%) HCWs and 53/65 (82%) microbiologists (Supplementary Tables 2 and 3). LP procedure kits and CSF analysis kits were also occasionally to always available as reported by 127/202 (63%) and 107/202 (53%) HCWs, respectively. Similarly, 42/65 (65%) microbiologists reported that CSF procedure kits were available.

LP pressure measurement tools had the lowest availability, with 134/202 (66%) HCWs rarely to never having access to it. CrAg titer measurement kits were also scarce, with 117/202 (57%) HCWs and 23/65 (35%) microbiologists reporting that they were rarely to never available.

Furthermore, CD4-determining machines were occasionally to always available according to only 151/202 (75%) HCWs and 60/65 (92%) microbiologists. Similarly, viral load–determining machines were occasionally to always available according to 131/202 (65%) HCWs and 49/65 (75%) microbiologists.

India ink reagents were reported as occasionally to always available by 50/65 (76%) microbiologist facilities, while 15/65 (24%) reported that they were rarely to never available. Lastly, HIV test kits were occasionally to always available according to 63/65 (96%) microbiologists.

### Treatment

#### Availability of Medications

**Figure 4.**
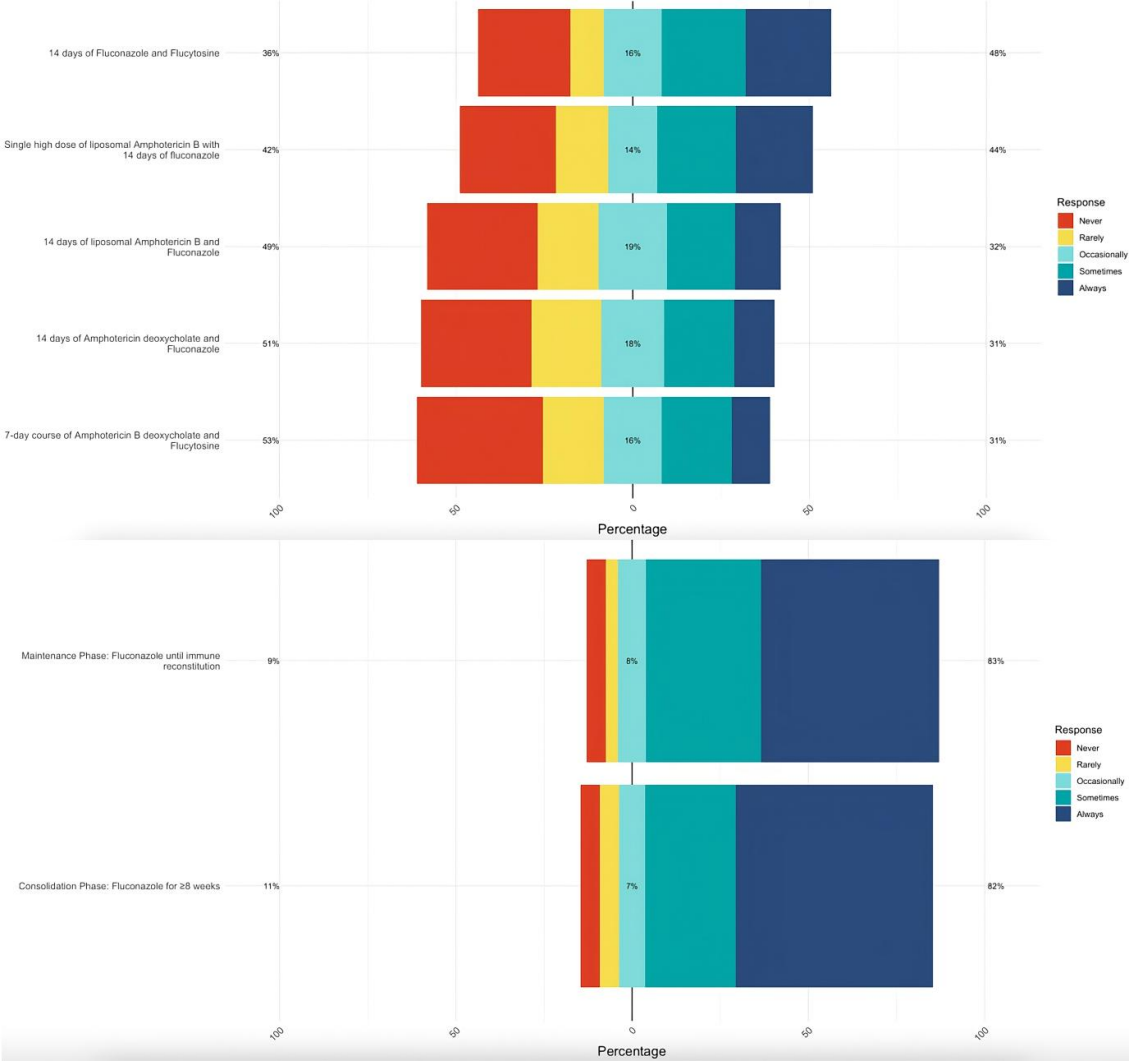
Available treatment according to HCWs. Abbreviation: HCWs, health care workers.

Among antifungals, fluconazole was relatively the most available, with 43/44 (97%) pharmacists and 130/202 (64%) HCWs reporting it as occasionally to always in stock (Figure 4; Supplementary Table 4). Amphotericin B deoxycholate was also occasionally to always available according to 14/44 (30%) pharmacists and 99/202 (49%) HCWs. Liposomal amphotericin B (L-Amb) was reported as rarely to never available, according to 19/44 (43%) pharmacists and 85/202 (43%) HCWs.

According to pharmacists, key factors limiting the use of these antifungals included limited availability, high cost, and low physician demand (Supplementary Table 5).

The most commonly available CM regimen was fluconazole for ≥8 weeks, with 180/202 (89%) HCWs indicating that it was occasionally to always available. Similarly, other regimens—such as single high-dose L-Amb plus 14 days of fluconazole, 7-day amphotericin B deoxycholate plus flucytosine, 14-day fluconazole plus flucytosine, and 14-day amphotericin B deoxycholate plus fluconazole—were each reported as occasionally to always available by 117/202 (57%), 95/202 (47%), 130/202 (64%), and 99/202 (49%) HCWs, respectively.

Compared with antifungals, the availability of additional treatments for CM, such as highly active antiretroviral therapy (HAART) and corticosteroids, was more consistent, with 40/44 (77%) and 43/44 (98%) pharmacists reporting them as occasionally to always available.

### Antifungal and Adjunctive Management

Initiating HAART at least 2 weeks after the diagnosis of CM was the most consistently performed in practice, with 193/202 (96%) HCWs occasionally to always prescribing it (Supplementary Table 6). Procedures to manage CSF pressure—such as lumbar puncture, ventriculostomy, ventriculoperitoneal (VP) shunt, or use of acetazolamide—were also occasionally to always performed according to 115/202 (56%) HCWs.

### Follow-up Measures

Daily clinical assessments during and after induction therapy were the most consistently performed follow-up measure, with 188/202 (93%) HCWs reporting that they were occasionally to always performed (Supplementary Table 7). Assessments for treatment failure (184/202 [91%]) and follow-up investigations such as LPs to exclude concomitant infections (169/202 [83%]) were also occasionally to always performed. Discontinuation of fluconazole after immune reconstitution was also occasionally to always performed by 171/202 (84%) HCWs. Furthermore, infectious disease consultations were occasionally to always conducted by 157/202 (77%) HCWs. Lastly, follow-up LPs on day 14 and repeat CrAg testing were occasionally to always performed, as reported by 136/202 (67%) and 127/202 (14%) HCWs, respectively.

### Barriers

A majority of HCWs (176/202 [87%]) reported that clinical guidelines for central nervous system (CNS) *Cryptococcus* infections are occasionally to always not well known, with 171/202 (84%) also citing limited availability as a barrier (Supplementary Table 8). Inadequate training programs were cited by 183/202 (91%) HCWs, and 169/202 (83%) mentioned the lack of effective feedback mechanisms. Language barriers and low public health awareness were also reported by 149/202 (70%) and 169/202 (83%) HCWs, respectively.

Regarding resources, the biggest barrier was the limited availability of ventriculostomy or VP shunt materials and neurosurgical expertise and decision support tools, as reported by 172/202 (85%) HCWs. This was followed by limited access to concise summaries (170/202 [84%]).

The most significant barriers to guideline implementation were insufficient policy support and challenges in integrating guidelines into electronic health records, as reported by 185/202 (92%) and 173/202 (85%) HCWs, respectively. Limited access to specialists was another major barrier, with 111/202 (55%) HCWs noting the unavailability of neurologists and 157/202 (77%) reporting difficulty accessing infectious disease consultants (Supplementary Table 9). On a positive note, 184/202 (91%) agreed that multidisciplinary teams improve adherence.

Clinical challenges included poor knowledge of intravenous L-Amb reconstitution (160/202 [79%]), workflow disruptions (172/202 [85%]), and patient-specific factors (176/202 [87%]). Inadequate follow-up mechanisms were noted by 185/202 (92%) HCWs. Economic, psychological, and social barriers were also significant: 190/202 (94%) cited economic considerations, 184/202 (91%) reported stress and burnout, and 171/202 (85%) were influenced by peer opinions.

## DISCUSSION

Assessing on-the-ground workflow, resources, and obstacles in CM care is crucial to narrowing the diagnostic and treatment gap and improving patient outcomes globally. Our work revealed significant variations in screening practices, resources, and access to antifungal medications across facilities in Ethiopia.

Early detection of HIV is not only a crucial entry point to HIV care but also provides a window for screening opportunistic infections—a cornerstone of CM diagnosis. While our study revealed that health care professionals generally perceive HIV test kits to be readily available, national reports indicate that the overall coverage of universal HIV testing in Ethiopia remains low, highlighting a disconnect between perceived and actual access [12, 13]. More importantly, HIV testing alone is not enough—it must be followed by CD4 count and viral load measurement. Our findings revealed limited access to these critical follow-up tests, which are essential for guiding appropriate treatment.

CrAg screening among PWH with CD4 counts <100 cells/mm³ is a key recommended strategy before HAART initiation [9]. However, our findings indicate that only 29% of health care workers routinely implement this practice. Similar low uptake has also been observed in other settings, such as Uganda, where reports indicate that only 20%–30% of eligible patients undergo CrAg screening [10]. In our work, this gap extended to diagnostic follow-through, with only 30% of HCWs performing an LP with CSF CrAg assay or India ink. Beyond clinical practice, these challenges are further compounded by our finding of reported limited availability of essential diagnostic tools and equipment, such as lateral flow assays, latex agglutination tests, India ink, LP, and CSF kits.

In terms of treatment, our study revealed that fluconazole was relatively more available, while guideline-based acute treatments such as L-Amb, amphotericin B deoxycholate, and flucytosine were less available. This reflects well-documented treatment challenges for CM in resource-limited settings worldwide, often related to high drug costs, restricted procurement channels, and inconsistent supply chains, particularly in resource-limited settings [14–16]. Furthermore, other essential interventions, such as timely HAART initiation, effective Intracranial pressure (ICP) management, CSF pressure regulation, ventriculostomy, and VP shunting, remain largely inaccessible, despite their role in reducing CM-related morbidity and mortality [17–19].

Beyond structural limitations, we also identified several perceived barriers to effective CM management. Many health care workers were unfamiliar with current guidelines due to limited access and inadequate training. This often results in inconsistent care practices, emphasizing the importance of accessible, up-to-date protocols. Studies have shown that adherence to standard treatment approaches for CM is associated with faster infection clearance and reduced mortality [20, 21]. Similar barriers have been reported in other low-resource settings, where limited infrastructure and logistical challenges compromise the delivery of CM-related services [10, 22, 23]. Insufficient policy support, lack of multidisciplinary teams, and low public awareness of CNS cryptococcosis remain significant barriers, greatly impacting disease outcomes. More importantly, psychosocial factors, including health care worker stress and peer influence, also contribute to suboptimal guideline adoption.

Overall, this resource scarcity is often stratified by level of health care facility, with academic and higher-tier hospitals typically having better access to essential diagnostic tools, while lower-level facilities face substantial limitations. This also became evident in our work when we divided the data by facility type (Supplementary Tables 10–18). Academic institutions like Hawassa and Wolaita had the best access to diagnostic tools, referral hospitals such as Zewditu and Amanuel showed moderate access to antifungals, and health centers like Felege Hiwot and Nifasilk Lafto faced the greatest limitations. Despite some differences, no substantial disparities were observed across facility types. These findings indicate that gaps are widespread rather than isolated to specific settings. However, the relatively small sample sizes within subgroups may have limited our ability to detect more subtle variations. Thus, this discordance between international guidelines and local reality within a country highlights how well-intentioned policies often falter without parallel investments in supply infrastructure and workforce training [9, 22, 23]. Hence, it is important to develop strategies that strengthen the alignment between international and local realities—going beyond contextual adaptation [24].

Several limitations of this study must be acknowledged. First, the reliance on self-reported practices from health care professionals introduces the potential for recall and social desirability biases, as respondents may have over- or underestimated their level of uptake of CM guidelines. Second, although the study included multiple facilities across Ethiopia, certain regions or facility types might remain underrepresented, limiting the overall generalizability of the findings. Third, the survey design captured data at a single time point and did not gather longitudinal or patient-outcome information, preventing the correlation of self-reported adoption with actual CM morbidity or mortality. Despite these constraints, the study's strengths include its multiprofessional scope, encompassing health care workers, pharmacists, and microbiologists, and its broad geographic sampling. This is, to our knowledge, the first large-scale survey of its kind in Ethiopia to integrate diagnostic, therapeutic, and barrier-related data on CM care.

Future research should focus on tracking patient outcomes alongside facility-level interventions, such as the introduction of consistent CrAg screening, to assess its impact on survival and relapse rates. Including patient data like CD4 counts and antiretroviral therapy status would clarify the clinical impact of the WHO guidelines. Studies should also examine the challenges in conflict-affected regions like Ayder Hospital, where supply disruptions and staffing shortages require targeted approaches. To improve CM care in Ethiopia, key steps include strengthening antifungal supply chains, scaling up CrAg screening for HIV patients with CD4 counts <200 cells/mm³, expanding training for health care workers, and using academic institutions to disseminate expertise. These actions can help address the burden of cryptococcal disease and improve outcomes for PWH.

## Supplementary Material

ofaf715_Supplementary_Data

## Supplementary Data

Supplementary materials are available at *Open Forum Infectious Diseases* online. Consisting of data provided by the authors to benefit the reader, the posted materials are not copyedited and are the sole responsibility of the authors, so questions or comments should be addressed to the corresponding author.
