# Supplementary material for: Assessment of Clinical Guideline Use in the Prevention, Diagnosis, and Treatment of Cryptococcal Meningitis Among Health Care Providers in Ethiopia
Source: Open Forum Infect Dis. 2025 Nov 24;12(12):ofaf715. doi: 10.1093/ofid/ofaf715 (PMC12687586; doi:10.1093/ofid/ofaf715)
Supplement: ofaf715_Supplementary_Data [file ofaf715_supplementary_data.docx]

**SUPPLEMENTARY**

**Table 1 Microbiologist Availability of diagnostic tests**

| How often do you use the following diagnostic methods with suspected Cryptococcal infections in your facility? | Never | Rarely | Occasionally | Sometimes | Always |
| --- | --- | --- | --- | --- | --- |
| 1. Confirmatory HIV test | 2 (3%) | 3 (5%) | 2 (3%) | 11 (17%) | 47 (72%) |
| 2. CD4 Determination | 3 (5%) | 1 (2%) | 2 (3%) | 32 (49%) | 27 (42%) |
| 3. Viral Load Measurement | 4 (6%) | 1 (2%) | 2 (3%) | 18 (28%) | 40 (62%) |
| 4. CSF Analysis | 8 (12%) | 7 (11%) | 4 (6%) | 30 (46%) | 16 (25%) |
| 5. CrAg test | 10 (15%) | 5 (8%) | 4 (6%) | 21 (32%) | 25 (39%) |
| 6. CrAg LFA | 15 (23%) | 7 (11%) | 4 (6%) | 22 (34%) | 17 (26%) |
| 7. CrAg Latex Agglutination Assay | 15 (23%) | 8 (12%) | 5 (8%) | 28 (43%) | 9 (14%) |
| 8. CSF India Ink | 14 (22%) | 5 (8%) | 5 (8%) | 20 (31%) | 21 (32%) |
| 9. Fungal culture | 19 (29%) | 5 (8%) | 8 (12%) | 24 (37%) | 9 (14%) |

**Table 2 HCW Availability of diagnostic tools/equipment**

| How often are this test tools/equipment available in your facility? | Never | Rarely | Occasionally | Sometimes | Always |
| --- | --- | --- | --- | --- | --- |
| 1. Cryptococcal Infection Guidelines | 30 (15%) | 16 (8%) | 25 (12%) | 69 (34%) | 62 (31%) |
| 2. LP procedure kits | 50 (25%) | 25 (12%) | 37 (18%) | 52 (26%) | 38 (18%) |
| 3. LP pressure measurement tool | 107(53%) | 27 (13%) | 32 (16%) | 23 (11%) | 13 (7%) |
| 4. CSF Analysis kit | 65 (32%) | 30 (15%) | 37 (18%) | 40 (20%) | 30 (15%) |
| 5. CrAg titer measurement kit | 77 (39%) | 37 (18%) | 42 (21%) | 31 (15%) | 15 (7%) |
| 6. CD4 determining machines | 33 (16%) | 18 (9%) | 25 (12%) | 62 (31%) | 64 (32%) |
| 7. Viral load determining machines | 54 (27%) | 17 (8%) | 32 (16%) | 58 (29%) | 41 (20%) |

**Table 3 Microbiologist Availability of diagnostic tools/equipment**

| How often are this test tools/equipment available in your facility? | Never | Rarely | Occasionally | Sometimes | Always |
| --- | --- | --- | --- | --- | --- |
| 1. Cryptococcal Infection Guidelines | 10 (15%) | 2 (3%) | 0 (0%) | 15 (23%) | 38 (59%) |
| 2. LP procedure kits | 15 (23%) | 4 (6%) | 2 (3%) | 25 (39%) | 19 (29%) |
| 3. CSF Reading kits | 17 (26%) | 6 (9%) | 2 (3%) | 21 (32%) | 19 (29%) |
| 4. CrAg Test kits | 19 (29%) | 4 (6%) | 4 (6%) | 22 (34%) | 16 (25%) |
| 5. Fungal Culture | 20 (31%) | 3 (5%) | 2 (3%) | 20 (31%) | 20 (31%) |
| 6. India Ink Reagents | 13 (20%) | 2 (3%) | 4 (6%) | 26 (40%) | 20 (31%) |
| 7. HIV Test kits | 1 (2%) | 1 (2%) | 5 (8%) | 24 (37%) | 34 (52%) |
| 8. CD4 Determination machines | 5 (8%) | 0 (0%) | 3 (5%) | 27 (42%) | 30 (46%) |
| 9. Viral load determination machines | 16 (25%) | 0 (0%) | 3 (5%) | 23 (35%) | 23 (35%) |

**Treatment**

**Table 4** **Available Treatment according to Pharmacists**

| How often are these medications for  cryptococcal meningitis treatment available in your facility? | Never | Rarely | Occasionally | Sometimes | Always |
| --- | --- | --- | --- | --- | --- |
| 1. Liposomal Amphotericin B | 12 (27%) | 7 (14%) | 5 (11%) | 14 (32%) | 7 (16%) |
| 2. Amphotericin deoxycholate | 24 (55%) | 6 (14%) | 2 (5%) | 12 (27%) | 0 (0%) |
| 3. Flucytosine | 19 (43%) | 7 (16%) | 5 (11%) | 7 (15%) | 6 (14%) |
| 4. Fluconazole | 1 (2%) | 0 (0%) | 2 (5%) | 20 (46%) | 21 (48%) |
| 5. HAART | 4 (9%) | 0 (0%) | 3 (7%) | 3 (7%) | 34 (77%) |
| 6. Corticosteroids | 1 (2%) | 0 (0%) | 2 (5%) | 12 (27%) | 29 (66%) |

**Table 5 Factors limiting the use of medications**

| What factors limit the use of liposomal amphotericin B at your facility | Limited availability of the medication | High cost, making it unaffordable for most patients | Low demand from physicians for this treatment | Current treatment guidelines do not recommend | Other reasons not listed | Use is not limited at my facility |
| --- | --- | --- | --- | --- | --- | --- |
| 1. Amphotericin deoxycholate | 25 (57%) | 4 (9%) | 9 (20%) | 3 (7%) | 2 (5%) | 1 (2%) |
| 2. Liposomal Amphotericin B | 27 (61%) | 6 (14%) | 4 (9%) | 1 (2%) | 1 (2%) | 5 (11%) |
| 3. Flucytosine | 21 (48%) | 5 (11%) | 8 (18%) | 3 (7%) | 3 (7%) | 4 (9%) |
| 4. Fluconazole | 23 (52%) | 1 (2%) | 6 (14%) | 1 (2%) | 2 (5%) | 11 (25%) |

**Anti-fungal and adjunctive management**

**Table 6 HCW Adjunctive management**

| How often are these non-pharmacological therapies for cryptococcal meningitis treatment available in your facility? | Never | Rarely | Occasionally | Sometimes | Always |
| --- | --- | --- | --- | --- | --- |
| 1. Routine use of adjunctive Corticosteroids during the induction phase | 35 (17%) | 18 (9%) | 23 (11%) | 81 (40%) | 45 (22%) |
| 2. HAART started after at least 2 weeks of diagnosis | 5 (3%) | 4 (2%) | 10 (5%) | 57 (28%) | 126 (62%) |
| 3. LP or lumbar drain, ventriculostomy, or VP shunt to maintain CSF pressure; 20 cmH2O | 51 (25%) | 36 (18%) | 36 (18%) | 53 (26%) | 26 (13%) |
| 4. Acetazolamide used to manage ICH | 62 (31%) | 38 (19%) | 36 (18%) | 42 (21%) | 24 (12%) |
| 5. Mannitol used for management of raised ICH | 37 (18%) | 29 (14%) | 33 (16%) | 45 (22%) | 58 (29%) |

**Follow-up**

**Table 7 HCW Follow up measures**

| How often are these follow-up measures performed for patients with cryptococcal meningitis? | Never | Rarely | Occasionally | Sometimes | Always |
| --- | --- | --- | --- | --- | --- |
| 1. Daily clinical response assessment during the initial two weeks of induction therapy and after completing induction therapy | 11 (5%) | 3 (2%) | 25 (12%) | 58 (29%) | 105 (52%) |
| 2. Infectious disease consultations | 29 (14%) | 16 (8%) | 25 (12%) | 69 (34%) | 63 (31%) |
| 3. Discontinuation of antifungal treatment if IRIS developed | 22 (11%) | 11 (5%) | 30 (15%) | 67 (33%) | 72 (36%) |
| 4. Routine follow-up LP at 14th day to assess antifungal treatment response | 41 (20%) | 25 (12%) | 39 (19%) | 68 (34%) | 29 (14%) |
| 5. Repeat serum or CSF CrAg to monitor the response | 50 (25%) | 25 (12%) | 37 (18%) | 62 (31%) | 28 (14%) |
| 6. Discontinuation of Fluconazole after immune reconstitution in those on ART with a CD4 count ≥200 cells/µL | 16 (8%) | 15 (7%) | 29 (14%) | 62 (31%) | 80 (39%) |
| 7. Assessments for underlying treatment failure in those with poor response | 9 (5%) | 9 (5%) | 29 (14%) | 65 (32%) | 90 (45%) |
| 8. LP and other relevant investigations to exclude concomitant infection in those with poor response to treatment | 21(10%) | 12 (6%) | 35 (17%) | 56 (28%) | 78 (39%) |
| 9. CSF prolonged fungal culture test for those with poor response to therapy | 69 (34%) | 20 (10%) | 42 (21%) | 42 (21%) | 33 (16%) |

**Barriers**

**Table 8 HCW Barriers to Cryptococcal Meningitis Guideline Implementation**

| **Barriers to Knowledge and Awareness of**  **Clinical Guidelines** | Never | Rarely | Occasionally | Sometimes | Always |
| --- | --- | --- | --- | --- | --- |
| The clinical guidelines for the treatment  of CNS Cryptococcus infections are not well-known among physicians | 10 (5%) | 16 (8%) | 35 (17%) | 106 (53%) | 35 (17%) |
| Limited availability of clinical  guidelines | 12 (5%) | 19 (9%) | 29 (14%) | 94 (46%) | 48 (23%) |
| Inadequate training programs on the use  of clinical guidelines contribute to their underutilization | 8 (4%) | 12 (6%) | 18 (9%) | 70 (34%) | 95 (47%) |
| Absence of effective feedback mechanisms  on guideline adherence | 9 (4%) | 24 (11%) | 31 (15%) | 75 (37%) | 63 (31%) |
| Understanding guidelines written in a  non-native language may affect their implementation | 27 (13%) | 33 (16%) | 42 (21%) | 71 (35%) | 29 (14%) |
| Level of public health awareness of CNS  Cryptococcus infections | 12 (6%) | 21 (10%) | 36 (17%) | 68 (33%) | 65 (32%) |
| Health workers' perception of the  efficacy and effectiveness of clinical guidelines influences their  willingness to adhere | 8 (4%) | 13 (6%) | 24 (11%) | 71 (35%) | 86 (43%) |
| **Barriers to Resources and Medications** |  |  |  |  |  |
| Limited availability of corticosteroids | 42(20%) | 38 (18%) | 36 (17%) | 70 (35%) | 16 (8%) |
| Limited availability of potassium chloride tablets | 25 (12%) | 37 (18%) | 34 (16%) | 83 (41%) | 23 (11%) |
| Limited availability of ventriculostomy or VP shunt procedures | 14 (7%) | 16 (7%) | 24 (12%) | 65 (32%) | 83 (41%) |
| Availability of concise summaries or quick-reference guides | 14 (7%) | 18 (8%) | 23 (11%) | 73 (36%) | 74 (37%) |
| Availability of decision support tools | 9 (4%) | 21 (10%) | 27 (13%) | 68 (33%) | 77 (38%) |
| **Barriers to Implementation and**  **Integration of Guidelines** |  |  |  |  |  |
| Insufficient policy support or alignment  with clinical guidelines affects their effective implementation | 3 (1%) | 14 (7%) | 33 (16%) | 84 (41%) | 68 (34%) |
| Integration of clinical guidelines into  electronic health records | 14 (7%) | 15 (7%) | 30 (15%) | 78 (38%) | 65 (32%) |
| Accessibility of timely updates and  revisions to clinical guidelines | 8 (4%) | 15 (16%) | 34 (16%) | 73 (36%) | 72 (36%) |

**Table 9 Healthcare System and Professional-Level Barriers**

| **Barriers to Healthcare Professionals** | Never | Rarely | Occasionally | Sometimes | Always |
| --- | --- | --- | --- | --- | --- |
| Neurologists are not available | 66 (32%) | 25 (12%) | 22 (11%) | 56 (38%) | 33 (16%) |
| Infectious disease consultants are not available | 22 (10%) | 23 (11%) | 25 (12%) | 55 (27%) | 77 (38%) |
| Neuroradiologists are not available | 30 (15%) | 10 (5%) | 16 (8%) | 40 (19%) | 106 (53%) |
| Presence of multidisciplinary healthcare teams positively affects adherence | 7 (3%) | 11 (5%) | 27 (13%) | 64 (31%) | 93 (46%) |
| **Clinical and Procedural Challenges** |  |  |  |  |  |
| Poor knowledge of the procedure for reconstituting intravenous L-Amb | 22 (10%) | 20 (9%) | 20 (9%) | 82 (40%) | 58 (28%) |
| Challenges in the daily workflow of physicians | 14 (7%) | 16 (8%) | 30 (15%) | 81 (40%) | 61 (30%) |
| Challenges related to patient-specific factors | 8 (4%) | 18 (9%) | 33 (16%) | 92 (45%) | 51 (25%) |
| Challenges in consultations among treating physicians | 16 (7%) | 26 (12%) | 34 (17%) | 81 (40%) | 45 (22%) |
| Inadequate mechanisms for patient follow-up | 6 (3%) | 11 (5%) | 31 (15%) | 86 (43%) | 68 (34%) |
| **Economic, Psychological, and Social Barriers** |  |  |  |  |  |
| Economic considerations, such as cost implications, affect physicians' practice | 3 (2%) | 9 (4%) | 23 (11%) | 73 (36%) | 94 (47%) |
| Psychosocial factors, such as stress or burnout, impact physicians | 8 (4%) | 10 (5%) | 37 (18%) | 73 (36%) | 74 (37%) |
| Influence of colleagues or peer physicians | 7 (3%) | 24 (12%) | 34 (17%) | 85 (42%) | 52 (26%) |

**SUBGROUP ANALYSIS BY FACILITY**

**Table 10. CM Screening and Prevention in Academic Institution**

| How often are these prevention and screening measures for Cryptococcal infections implemented in your facility? | Never | Rarely | Occasionally | Sometimes | Always |
| --- | --- | --- | --- | --- | --- |
| 1. Screening for cryptococcal antigen (CrAg) performed for patients with a CD4 count less than 100? | 11 (10%) | 11 (10%) | 17 (16%) | 33 (31%) | 35 (33%) |
| 2. Clinical evaluation for meningitis for people with HIV (PWH) who have a positive CrAg screening | 8 (8%) | 3 (2%) | 11 (10%) | 18 (17%) | 67 (63%) |
| 3. Lumbar puncture (LP) and India ink stain of CSF for PWH who have a positive serum CrAg screening | 10 (9%) | 4 (3%) | 14 (13%) | 37 (35%) | 42 (39%) |
| 4. Lumbar puncture (LP) and CrAg assay of CSF for PWH who have a positive serum CrAg screening | 11 (10%) | 7 (6%) | 15 (14%) | 39 (36%) | 35 (33%) |
| 5. Fluconazole prophylaxis given to PWH with a CD4 count of less than 100 when CrAg screening unavailable | 9 (8%) | 8 (7%) | 16 (15%) | 31 (29%) | 43 (40%) |

**Table 11. CM Screening and Prevention in Hospital**

| How often are these prevention and screening measures for Cryptococcal infections implemented in your facility? | Never | Rarely | Occasionally | Sometimes | Always |
| --- | --- | --- | --- | --- | --- |
| 1. Screening for cryptococcal antigen (CrAg) performed for patients with a CD4 count less than 100? | 8 (18%) | 3 (6%) | 5 (11%) | 12 (27%) | 16 (36%) |
| 2. Clinical evaluation for meningitis for people with HIV (PWH) who have a positive CrAg screening | 6 (7%) | 4 (9%) | 5 (11%) | 8 (18%) | 24 (55%) |
| 3. Lumbar puncture (LP) and India ink stain of CSF for PWH who have a positive serum CrAg screening | 7 (14%) | 5 (11%) | 5 (11%) | 10 (22%) | 17 (39%) |
| 4. Lumbar puncture (LP) and CrAg assay of CSF for PWH who have a positive serum CrAg screening | 6 (13%) | 5 (11%) | 7 (16%) | 9 (21%) | 17 (39%) |
| 5. Fluconazole prophylaxis given to PWH with a CD4 count of less than 100 when CrAg screening unavailable | 2 (4%) | 5 (11%) | 4 (9%) | 10 (22%) | 23 (52%) |

**Table 12. CM Screening and Prevention in Health Center**

| How often are these prevention and screening measures for Cryptococcal infections implemented in your facility? | Never | Rarely | Occasionally | Sometimes | Always |
| --- | --- | --- | --- | --- | --- |
| 1. Screening for cryptococcal antigen (CrAg) performed for patients with a CD4 count less than 100? | 15 (35%) | 8 (19%) | 3 (7%) | 10 (23%) | 6 (14%) |
| 2. Clinical evaluation for meningitis for people with HIV (PWH) who have a positive CrAg screening | 7 (16%) | 8 (19%) | 4 (9%) | 10 (23%) | 13 (31%) |
| 3. Lumbar puncture (LP) and India ink stain of CSF for PWH who have a positive serum CrAg screening | 18 (42%) | 9 (21%) | 3 (7%) | 9 (21%) | 3 (7%) |
| 4. Lumbar puncture (LP) and CrAg assay of CSF for PWH who have a positive serum CrAg screening | 19 (45%) | 10 (23%) | 2 (4%) | 6 (14%) | 5 (12%) |
| 5. Fluconazole prophylaxis given to PWH with a CD4 count of less than 100 when CrAg screening unavailable | 5 (11%) | 5 (11%) | 3 (7%) | 18 (42%) | 11 (26%) |

**Table 13 CM Diagnosis in Academic Institution**

| How often are these diagnostic tests for cryptococcal infections available at your facility? | Never | Rarely | Occasionally | Sometimes | Always |
| --- | --- | --- | --- | --- | --- |
| 1. HIV test | 0 (0%) | 1 (1%) | 2 (2%) | 32 (30%%) | 72 (67%) |
| 2. CD4 Determination | 5 (4%) | 5 (4%) | 10 (9%) | 52 (49%) | 34 (32%) |
| 3. Viral Load Measurement | 20 (19%) | 12 (11%) | 12 (11%) | 44 (41%) | 18 (17%) |
| 4. CSF Analysis | 11 (10%) | 10 (9%) | 20 (19%) | 34 (32%) | 31 (29%) |
| 5. LP and CSF pressure measurement | 52 (49%) | 8 (7%) | 17 (16%) | 15 (14%) | 15 (14%) |
| 6. Lateral flow or Latex agglutination | 50 (46%) | 12 (11%) | 21 (19%) | 20 (18%) | 4 (4%) |
| 7. CrAg (Latex Agglutination Assay) | 46 (43%) | 10 (9%) | 26 (24%) | 17 (16%) | 6 (6%) |
| 8. CSF India Ink | 26 (24%) | 11 (10%) | 19 (17%) | 33 (30%) | 18 (17%) |
| 9. Rapid serum plasma or whole blood | 32 (30%) | 13 (12%) | 25 (23%) | 28 (26%) | 9 (8%) |
| 10. Fungal culture | 53 (50%) | 10 (9%) | 18 (17%) | 20 (19%) | 5 (4%) |
| 11. Brain CT scan | 4 (3%) | 0 (0%) | 8 (8%) | 26 (24%) | 69 (65%) |
| 12. Brain MRI | 6 (6%) | 2 (2%) | 14 (13%) | 48 (44%) | 37 (35%) |

**Table 14. CM Diagnosis in Hospital**

| How often are these diagnostic tests for cryptococcal infections available at your facility? | Never | Rarely | Occasionally | Sometimes | Always |
| --- | --- | --- | --- | --- | --- |
| 1. HIV test | 1 (2%) | 0 (0%) | 1 (2%) | 5 (11%%) | 37 (84%) |
| 2. CD4 Determination | 4 (9%) | 3 (6%) | 3 (6%) | 10 (23%) | 24 (54%) |
| 3. Viral Load Measurement | 6 (14%) | 2 (4%) | 5 (11%) | 11 (26%) | 18 (43%) |
| 4. CSF Analysis | 8 (18%) | 5 (12%) | 8 (18%) | 11 (26%) | 11 (26%) |
| 5. LP and CSF pressure measurement | 15 (35%) | 4 (9%) | 8 (18%) | 6 (14%) | 10 (23%) |
| 6. Lateral flow or Latex agglutination | 17 (39%) | 5 (11%) | 8 (18%) | 6 (13%) | 8 (18%) |
| 7. CrAg (Latex Agglutination Assay) | 16 (37%) | 5 (11%) | 8 (18%) | 9 (20%) | 5 (12%) |
| 8. CSF India Ink | 13 (30%) | 6 (14%) | 9 (21%) | 4 (9%) | 11 (25%) |
| 9. Rapid serum plasma or whole blood | 13 (30%) | 6 (14%) | 10 (23%) | 7 (16%) | 7 (16%) |
| 10. Fungal culture | 18 (41%) | 6 (13%) | 10 (23%) | 5 (11%) | 5 (11%) |
| 11. Brain CT scan | 23 (52%) | 2 (5%) | 8 (18%) | 4 (9%) | 7 (16%) |
| 12. Brain MRI | 23 (52%) | 2 (4%) | 8 (18%) | 6 (13%) | 5 (11%) |

**Table 15. CM Diagnosis in Health center**

| How often are these diagnostic tests for cryptococcal infections available at your facility? | Never | Rarely | Occasionally | Sometimes | Always |
| --- | --- | --- | --- | --- | --- |
| 1. HIV test | 1 (2%) | 0 (0%) | 1 (2%) | 4 (10%%) | 35 (85%) |
| 2. CD4 Determination | 2 (5%) | 0 (0%) | 2 (5%) | 11 (27%) | 26 (63%) |
| 3. Viral Load Measurement | 3 (7%) | 1 (3%) | 1 (3%) | 9 (22%) | 26 (65%) |
| 4. CSF Analysis | 18 (45%) | 9 (22%) | 6 (15%) | 6 (15%) | 1 (3%) |
| 5. LP and CSF pressure measurement | 19 (47%) | 9 (22%) | 5 (12%) | 7 (17%) | 0 (0%) |
| 6. Lateral flow or Latex agglutination | 16 (41%) | 10 (25%) | 5 (13%) | 6 (15%) | 2 (5%) |
| 7. CrAg (Latex Agglutination Assay) | 17 (42%) | 12 (30%) | 4 (10%) | 6 (15%) | 2 (2%) |
| 8. CSF India Ink | 13 (31%) | 10 (24%) | 3 (7%) | 8 (20%) | 7 (17%) |
| 9. Rapid serum plasma or whole blood | 18 (46%) | 11 (28%) | 5 (12%) | 3 (7%) | 2 (5%) |
| 10. Fungal culture | 14 (34%) | 13 (31%) | 6 (14%) | 5 (12%) | 3 (7%) |
| 11. Brain CT scan | 20 (50%) | 7 (17%) | 7 (17%) | 4 (10%) | 2 (5%) |
| 12. Brain MRI | 20 (51%) | 7 (17%) | 6 (15%) | 5 (12%) | 1 (3%) |

**Table 16 CM Treatment in Academic institution**

| What is the standard induction treatment for cryptococcal meningitis available in your facility? | Never | Rarely | Occasionally | Sometimes | Always |
| --- | --- | --- | --- | --- | --- |
| 1. A single high dose of liposomal Amphotericin B with 14 days of and fluconazole | 31 (29%) | 8 (8%) | 16 (15%) | 28 (26%) | 24 (22%) |
| 2. A 7-day course of Amphotericin B deoxycholate and Flucytosine | 43 (40%) | 11 (10%) | 20 (18%) | 21 (20%) | 12 (11%) |
| 3. 14 days of Fluconazole and Flucytosine | 32 (29%) | 10 (9%) | 17 (15%) | 23 (21%) | 25 (23%) |
| 4. 14 days of liposomal Amphotericin B and Fluconazole | 31 (29%) | 17 (15%) | 22 (20%) | 24 (22%) | 13 (12%) |
| 5. 14 days of Amphotericin deoxycholate and Fluconazole | 33 (31%) | 18 (16%) | 20 (18%) | 24 (22%) | 12 (11%) |

**Table 17 CM Treatment in Hospital**

| What is the standard induction treatment for cryptococcal meningitis available in your facility? | Never | Rarely | Occasionally | Sometimes | Always |
| --- | --- | --- | --- | --- | --- |
| 1. A single high dose of liposomal Amphotericin B with 14 days of and fluconazole | 9 (21%) | 8 (18%) | 5 (11%) | 9 (21%) | 13 (30%) |
| 2. A 7-day course of Amphotericin B deoxycholate and Flucytosine | 12 (27%) | 10 (22%) | 7 (15%) | 10 (22%) | 5 (11%) |
| 3. 14 days of Fluconazole and Flucytosine | 10 (23%) | 5 (11%) | 6 (14%) | 11 (25%) | 12 (27%) |
| 4. 14 days of liposomal Amphotericin B and Fluconazole | 16 (36%) | 7 (15%) | 7 (15%) | 9 (21%) | 5 (11%) |
| 5. 14 days of Amphotericin deoxycholate and Fluconazole | 16 (36%) | 9 (20%) | 6 (14%) | 9 (20%) | 4 (9%) |

**Table 18 CM Treatment in Health Center**

| What is the standard induction treatment for cryptococcal meningitis available in your facility? | Never | Rarely | Occasionally | Sometimes | Always |
| --- | --- | --- | --- | --- | --- |
| 1. A single high dose of liposomal Amphotericin B with 14 days of and fluconazole | 13 (31%) | 11 (26%) | 5 (12%) | 7 (17%) | 6 (14%) |
| 2. A 7-day course of Amphotericin B deoxycholate and Flucytosine | 13 (31%) | 11 (26%) | 5 (12%) | 8 (19%) | 5 (12%) |
| 3. 14 days of Fluconazole and Flucytosine | 6 (14%) | 1 (2%) | 10 (23%) | 14 (33%) | 11 (26%) |
| 4. 14 days of liposomal Amphotericin B and Fluconazole | 13 (31%) | 10 (23%) | 7 (16%) | 6 (14%) | 6 (14%) |
| 5. 14 days of Amphotericin deoxycholate and Fluconazole | 11 (26%) | 10 (23%) | 8 (19%) | 7 (16%) | 6 (14%) |

**SURVEY MATERIAL**

**Health Care Worker Survey**

**Questionnaire on ‘’Assessment of Adherence to Clinical Guideline Use in the Prevention, Diagnosis, and Treatment of CNS Cryptococcus Infections According to WHO 2022 Guidelines, among Physicians and other Healthcare Providers in Ethiopia’’**

I kindly request your participation in a survey entitled "’ Assessment of Adherence to Clinical Guideline Use in the Prevention, Diagnosis, and Treatment of CNS Cryptococcus Infections According to WHO 2022 Guidelines, among Physicians and other Healthcare Providers in Ethiopia’’. Your expertise and insights are of great value in our survey.

The purpose of this survey is to identify the barriers that healthcare providers face to the effective implementation of clinical guidelines in the treatment of CNS Cryptococcus infections. By understanding these challenges, we aim to improve the quality of care and outcomes for patients with this condition. Your contribution to this study will greatly assist us in capturing a comprehensive perspective on the current state of guideline adherence in Africa ultimately leading to the development of targeted interventions and strategies to improve clinical practice in the region.

The survey questionnaire has been designed to gather information about your experiences, perceptions, and specific challenges faced in practicing guideline recommendations in the context of CNS Cryptococcus infections. Your responses will remain completely anonymous and confidential, ensuring that your individual views are protected. The survey will take approximately 10-15 minutes to complete.

Should you choose to participate, please find the link to the survey questionnaire. We kindly ask that you submit your responses no later than the 10^th^ of July to ensure that your input can be included in the analysis phase.

On behalf of the research team, I would like to express our sincerest gratitude for considering our request to participate in this important study. Your expertise and feedback are invaluable to us, and we highly value the time and effort you will dedicate to completing the survey.

Should you require any further information or have any questions about the study, please do not hesitate to contact me at [mamoblen83@gmail.com](mailto:mamoblen83@gmail.com)

**Section I**

**Demographic data**

1. On average how many patients with suspected HIV-associated CNS cryptococcus infection have you encountered in the past year?

A. 0

B. 1-10

C. 10-20

D. 20-30

E. 30-40

F. More than 50

2. Please select your role

A. Neurologist

B. Infectious diseases physicians

C. Internist

D. Emergency medicine physician

D. Neurology resident

E. Emergency medicine resident

F. Internal medicine resident

G. General practitioner

H. Other, specify………………….

3. How many years of experience do you have in working with patients who may have CNS Cryptococcal infection?

1. <1 year
2. 1-5 years
3. 5-10 years
4. > 10 years

4. Please indicate the type of your primary practice:

A. Academic institution

B. Referral hospital

C. District hospital

D. Private practice

5. Please mention the name of your center

………………………………………………………………………………………………………

6. Where is your primary practice located

A. Within a major city

B. Sub-urban

C. moderate-sized city

D. Small City

E. Rural area

7. Which international guideline informs your approach to cryptococcal disease prevention, diagnosis, and management? (Please attach the link or the pdf document)

....................................................................................................................…………………

8. What is the latest national cryptococcal disease prevention, diagnosis, and management guideline you use as a reference

………………………………………………………………………………………………….

9. Are you aware of the 2022 WHO Cryptococcal diagnosis and treatment guidelines?

A. Yes

B. No

9. Have you received training on the 2022 WHO cryptococcal infection prevention, diagnosis, and treatment guidelines?

A. No

B. Yes

11. How easily can you access updated Cryptococcus prevention, diagnosis, and treatment guidelines?

A. Very easily

B. Easily

C. Somewhat difficult

D. Difficult

E. Very difficult

12. On average how many of your colleagues are aware of Cryptococcal prevention, diagnosis, and treatment guidelines?

A. 100%

B. 50%-100%

C. 25%-50%

D. 0%-25%

13. Do you believe there is a need for additional training related to Cryptococcal prevention, diagnosis, and treatment for health workers?

A. Yes

B. No

**Section II**

**Assessment of knowledge on the 2022 WHO Cryptococcus prevention, diagnosis, and management guidelines, based on the 2018 EQUAL Cryptococcus score**

1. **EQUAL Cryptococcus Score 2018:** a simple tool to summarize guideline recommendations that could be used to evaluate guideline adherence as a marker of quality of care and to support antimicrobial stewardship.

| **Prevention and Screening**  **How often are these** **prevention and screening measures and tests for Cryptococcal infections available in your facility?** | | | | | | |
| --- | --- | --- | --- | --- | --- | --- |
|  | | **Always** | **Sometimes** | **Occasionally** | **Rarely** | **Never** |
| - - - 1. How often is screening for plasma, serum, or whole blood cryptococcal antigen performed for patients with a CD4 count less than 100? | |  |  |  |  |  |
| 1. For People Living with HIV (PLWH) who have a positive Crag screening, how often are they evaluated for meningitis and undergo lumbar puncture (LP) with CSF Indian ink or Crag? | |  |  |  |  |  |
| 1. When Crag screening is not available, how often is fluconazole primary prophylaxis given for People Living with HIV with a CD4 count less than 100? | |  |  |  |  |  |
| **Diagnosis**  **How often are these** **diagnostic tests for Cryptococcal infections available at your facility?** | | | | | | |
|  | | **Always** | **Sometimes** | **Occasionally** | **Rarely** | **Never** |
| **1.** | HIV tests |  |  |  |  |  |
| **2.** | CD4 determination |  |  |  |  |  |
| **3.** | Viral load measurement |  |  |  |  |  |
| **4.** | CSF analysis |  |  |  |  |  |
| **5.** | LP and CSF pressure measurement |  |  |  |  |  |
| **5.** | Rapid CSF Cryptococcal Antigen test |  |  |  |  |  |
| 6. | Cryptococcal Antigen assay (lateral **flow assay**) |  |  |  |  |  |
| 7. | Cryptococcal Antigen assay **(latex agglutination assay)** |  |  |  |  |  |
| **8.** | CSF India ink test |  |  |  |  |  |
| **9.** | Rapid **serum** plasma or whole blood cryptococcal antigen kits |  |  |  |  |  |
| **10** | Fungal culture |  |  |  |  |  |
| **11** | Brain CT scan |  |  |  |  |  |
| **12** | Brain MRI |  |  |  |  |  |
|  | **How often are these test tools or equipment available in your facility?** | **Always** | **Sometimes** | **Occasionally** | **Rarely** | **Never** |
| **1.** | Availability of cryptococcal infection guidelines |  |  |  |  |  |
| **2.** | Availability of LP procedure kits |  |  |  |  |  |
| **3.** | Availability of LP pressure measurement tool |  |  |  |  |  |
| **4.** | Availability of CSF reading kit |  |  |  |  |  |
| **5.** | Availability of CSF Cryptococcal Ag titer measurement kit |  |  |  |  |  |
| **6.** | Availability of fungal culture kit |  |  |  |  |  |
| **7.** | Availability of CSF India ink kit |  |  |  |  |  |
| **8.** | Availability of HIV test kits |  |  |  |  |  |
| **9.** | Availability of CD4-determining machines |  |  |  |  |  |
| **10** | Availability of Viral load- determining machines |  |  |  |  |  |
| **Antifungal treatment**  **How often are these medications for cryptococcal meningitis treatment available in your facility?** | | | | | | |
|  | **Induction phase** | **Always** | **Sometimes** | **Occasionally** | **Rarely** | **Never** |
| **1.** | A single high dose of **liposomal** Amphotericin B with 14 days of and **fluconazole** |  |  |  |  |  |
| **2.** | A 7-day course of **Amphotericin B deoxycholate** and **Flucytosine** |  |  |  |  |  |
| **3.** | 14 days of **Fluconazol**e and **Flucytosine** |  |  |  |  |  |
| **4.** | 14 days of **liposomal Amphotericin B** and **Fluconazole** |  |  |  |  |  |
| **5.** | 14 days of **Amphotericin deoxycholate** and **Fluconazole** |  |  |  |  |  |
| **6. If the listed options are not the standard protocols in your region, what induction regimen is typically used for patients with cryptococcal meningitis?**  **………………………………………………………………………………………………………………………..** | | | | | | |
|  | **Consolidation phase** | **Always** | **Sometimes** | **Occasionally** | **Rarely** | **Never** |
| **7.** | Fluconazole for ≥8 week |  |  |  |  |  |
| **8. If the listed option is not the standard protocols in your region, what consolidation regimen is typically used for patients with cryptococcal meningitis?**  **………………………………………………………………………………………………………………………..** | | | | | | |
|  | **Maintenance phase** | **Always** | **Sometimes** | **Occasionally** | **Rarely** | **Never** |
| **9.** | Fluconazole until immune reconstitution (CD4 >200 cells/mm3) and suppression of viral loads on ART |  |  |  |  |  |
| **10. If the listed options are not the standard protocols in your region, what maintenance regimen is typically used for patients with cryptococcal meningitis?**  **………………………………………………………………………………………………………………………..** | | | | | | |
| **Non-pharmacological therapeutic interventions** | | | | | | |
|  | How often are these non-pharmacology therapies used for Cryptococcal meningitis? | **Always** | **Sometimes** | **occasionally** | **Rarely** | **Never** |
| **1** | Routine use of adjunctive Corticosteroids during the induction phase |  |  |  |  |  |
| **2** | HAART started **after** at least 2 weeks of diagnosis |  |  |  |  |  |
| **3** | LP or lumbar drain, ventriculostomy, or VP shunt to maintain CSF pressure <20 cmH2O |  |  |  |  |  |
| **4** | Acetazolamide used to manage ICH |  |  |  |  |  |
| **5** | Mannitol used for management of raised ICH |  |  |  |  |  |
| **Follow up** | | | | | | |
|  | **How often are these follow-up measures performed for patients with cryptococcal meningitis?** | **Always** | **Sometimes** | **Occasionally** | **Rarely** | **Never** |
| **1** | Daily clinical response assessment during the initial two weeks of induction therapy and after completing induction therapy |  |  |  |  |  |
| **2** | Infectious disease consultations |  |  |  |  |  |
| **3** | Discontinuation of antifungal treatment if IRIS developed? |  |  |  |  |  |
| **4** | Routine follow-up LP at 14th day to assess antifungal treatment response |  |  |  |  |  |
| **5** | Repeat **serum or CSF** CrAg to monitor the response |  |  |  |  |  |
| **6** | Discontinuation of Fluconazole after immune reconstitution in those on ART with a CD4 count ≥200 cells/µL |  |  |  |  |  |
| **7** | Assessments for underlying treatment failure in those with poor response. |  |  |  |  |  |
| **8** | LP and other relevant investigations to exclude concomitant infection in those with poor response to treatment. |  |  |  |  |  |
| **9** | CSF prolonged fungal culture test for those with poor response to therapy. |  |  |  |  |  |
| **To what extent do you agree with the following statements regarding barriers to following guidelines for cryptococcal meningitis treatment?** | | | | | | |
|  |  | **Strongly agree** | **Agree** | **Neutral** | **Disagree** | **Strongly disagree** |
| **1** | The clinical guidelines for the treatment of CNS Cryptococcus infections are not well-known among physicians. |  |  |  |  |  |
| **2** | There is limited availability of clinical guidelines |  |  |  |  |  |
| **3** | There is limited availability of corticosteroids |  |  |  |  |  |
| **4** | There is limited availability of potassium chloride tablets |  |  |  |  |  |
| **5** | There is limited availability of ventriculostomy or VP shunt procedures |  |  |  |  |  |
| **6** | Neurologists are not available in your setup |  |  |  |  |  |
| **7** | Infectious disease consultants are not available in your setup |  |  |  |  |  |
| **8** | Neuroradiologists are not available in your setup |  |  |  |  |  |
| **9** | Inadequate training programs on the use of clinical guidelines contribute to their underutilization |  |  |  |  |  |
| **10** | Poor knowledge of the procedure for reconstituting intravenous Liposomal Amphotericin B contribute to its underutilization |  |  |  |  |  |
| **11** | Challenges in the daily workflow of physicians make it difficult to incorporate clinical guidelines into practice |  |  |  |  |  |
| **12** | Challenges related to patient-specific factors, such as complex cases, impede adherence to clinical guidelines |  |  |  |  |  |
| **13** | Challenges in consultations among treating physicians hinder the effective use of clinical guidelines. |  |  |  |  |  |
| **14** | The absence of effective feedback mechanisms on guideline adherence prevents continuous improvement. |  |  |  |  |  |
| **15** | Insufficient policy support or alignment with clinical guidelines affects their effective implementation. |  |  |  |  |  |
| **16** | The availability of continuing education opportunities specifically addressing CNS infections impacts physicians' adherence to clinical guidelines. |  |  |  |  |  |
| **17** | The availability of concise summaries or quick-reference guides for clinical guidelines affects their practical use. |  |  |  |  |  |
| **18** | Inadequate mechanisms for patient follow-up contribute to the ineffective application of clinical guidelines. |  |  |  |  |  |
| **19** | Economic considerations, such as cost implications affect physicians' practice. |  |  |  |  |  |
| **20** | The integration of clinical guidelines into electronic health records impacts their practical utility. |  |  |  |  |  |
| **21** | Psychosocial factors, such as stress or burnout, impact physicians' ability to follow clinical guidelines |  |  |  |  |  |
| **22** | The accessibility of timely updates and revisions to clinical guidelines impacts their relevance and applicability. |  |  |  |  |  |
| **23** | The influence of colleagues or peer physicians in following clinical guidelines affects individual adherence. |  |  |  |  |  |
| **24** | Understanding guidelines written in a non-native language may affect their implementation. |  |  |  |  |  |
| **25** | The level of public health awareness of CNS Cryptococcus infections influences guideline adherence. |  |  |  |  |  |
| **26** | The availability of decision support tools, such as algorithms or decision trees, facilitates guideline adherence. |  |  |  |  |  |
| **27** | The presence of multidisciplinary healthcare teams positively affects adherence to guidelines |  |  |  |  |  |
| **28** | Health workers' perception of the efficacy and effectiveness of clinical guidelines influences their willingness to adhere. |  |  |  |  |  |

29. Do you have any additional feedback or comments that you would like to share that are not covered in the questionnaire? If so, please share them with us….………………………………………………………………………………………………………………………………………………………………………………………………………………………………………………………………………………………………………………

**Microbiologists Survey**

**Questionnaire on ‘’Assessment of Adherence to Clinical Guideline Use in the Prevention, Diagnosis, and Treatment of CNS Cryptococcus Infections According to WHO 2022 Guidelines, among Physicians and other Healthcare Providers in Ethiopia’’**

I kindly request your participation in a survey entitled "’ Assessment of Adherence to Clinical Guideline Use in the Prevention, Diagnosis, and Treatment of CNS Cryptococcus Infections According to WHO 2022 Guidelines, among Physicians and other Healthcare Providers in Ethiopia’’. Your expertise and insights are of great value in our survey.

The purpose of this survey is to identify the barriers that healthcare providers face to the effective implementation of clinical guidelines in the treatment of CNS Cryptococcus infections. By understanding these challenges, we aim to improve the quality of care and outcomes for patients with this condition. Your contribution to this study will greatly assist us in capturing a comprehensive perspective on the current state of guideline adherence in Africa ultimately leading to the development of targeted interventions and strategies to improve clinical practice in the region.

The survey questionnaire has been designed to gather information about your experiences, perceptions, and specific challenges faced in practicing guideline recommendations in the context of CNS Cryptococcus infections. Your responses will remain completely anonymous and confidential, ensuring that your individual views are protected. The survey will take approximately 10-15 minutes to complete.

We kindly ask that you submit your responses within two weeks of being sent this survey for data analysis. All responses will be accepted if submitted later.

On behalf of the research team, I would like to express our sincerest gratitude for considering our request to participate in this important study. Your expertise and feedback are invaluable to us, and we highly value the time and effort you will dedicate to completing the survey.

Should you require any further information or have any questions about the study, please do not hesitate to contact me at [mamoblen83@gmail.com](mailto:mamoblen83@gmail.com)

**Section I**

**Demographic Data**

1. On average how many cases of suspected HIV-associated CNS Cryptococcus infection have you encountered in the past year?
   - Less than 5 cases
   - 5-20 cases
   - Over 20 cases per year
2. What is your title or position within the microbiology laboratory?

…………………………………………………………………………………………………………………..

1. How long have you been involved in microbiological diagnosis of Cryptococcal infection diagnosis?
   - <1 year
   - 1-5 years
   - 6-10 years
   - >10 years
2. Please indicate the type of your primary practice:
   - Academic Institution
   - Referral hospital
   - District hospital
   - Private practice
3. Where is your primary practice located?
   - Metropolis (population >1,000,000)
   - City (population >100,000 to 1,000,000)
   - Large town (population >50,000 to 100,000)
   - Medium town (population >20,000 to 50,000)
   - Small town (population 2000 to 20,000)
4. Please mention the name of your center:

…………………………………………………………………………………………………………………………….

1. Which guideline/SOP informs your approach to cryptococcal infection diagnosis?

…………………………………………………………………………………………………………………………….

1. Please attach pdf document or the link of the guidelines  referenced above if accessible (Hospital/Regional/National/WHO guidelines you use in your facility if you have access)

…………………………………………………………………………………………………………………………….

1. Are you aware of the 2022 WHO Cryptococcal prevention, diagnosis, and treatment guidelines?
   - Yes
   - No
2. On average, how many of your colleagues are aware of the 2022 WHO cryptococcal diagnosis guidelines?
   - 100%
   - 76-99%
   - 51-75%
   - 26-50%
   - 0-25%
3. Have you received training on the 2022 WHO cryptococcal infection diagnosis guidelines?
   - Yes
   - No
4. If your answer is yes to the above question, What was the date of your training?

--/--/----

1. Do you believe there is a need for additional training related to cryptococcal disease diagnosis for Microbiologists?
   - Yes
   - No

**Section II**

**EQUAL Cryptococcus Score 2018**

A simple tool to summarize guideline recommendations that could be used to evaluate guideline adherence as a marker of quality of care and to support antimicrobial stewardship. Please select the most accurate option.

1. How often do you use the following diagnostic methods with suspected cryptococcal infections?

|  | Always | Sometimes | Occasionally | Rarely | Never |
| --- | --- | --- | --- | --- | --- |
| 1. Confirmatory HIV Tests |  |  |  |  |  |
| 2. CD4 determination |  |  |  |  |  |
| 3. Viral load measurement |  |  |  |  |  |
| 4. CSF analysis |  |  |  |  |  |
| 5. Rapid CSF cryptococcal antigen test (CrAg) |  |  |  |  |  |
| 6. Cryptococcal Antigen assay (lateral flow assay) |  |  |  |  |  |
| 7. Cryptococcal Antigen assay (latex agglutination assay) |  |  |  |  |  |
| 8. CSF India Ink test |  |  |  |  |  |
| 9. Rapid serum plasma or whole blood cryptococcal antigen kits |  |  |  |  |  |
| 10. Fungal culture |  |  |  |  |  |

1. How often are these test tools/equipment available in your facility?

|  | Always | Sometimes | Occasionally | Rarely | Never |
| --- | --- | --- | --- | --- | --- |
| 1. Cryptococcal diagnostic guidelines |  |  |  |  |  |
| 2. LP procedure kits |  |  |  |  |  |
| 3. CSF reading kits |  |  |  |  |  |
| 4. CSF cryptococcal Ag titer measurement kit |  |  |  |  |  |
| 5. Fungal culture equipment |  |  |  |  |  |
| 6. CSF India ink test |  |  |  |  |  |
| 7. HIV test kits |  |  |  |  |  |
| 8. CD4-determining machines |  |  |  |  |  |
| 9. Viral load-determining machines |  |  |  |  |  |

**Section III**

Assessment of barriers to guideline adherence: Please rate the following statements on a scale from "**Strongly Disagree**" to "**Strongly Agree**."

To what extent do you agree with the following statements regarding barriers to following guidelines for cryptococcal meningitis diagnosis?

|  | Strongly Agree | Agree | Neutral | Disagree | Strongly Disagree |
| --- | --- | --- | --- | --- | --- |
| 1. The clinical guidelines for the diagnosis of CNS Cryptococcus infections are not well-known among laboratory technologists. |  |  |  |  |  |
| 2. Challenges in consultations with treating physicians hinder the effective use of clinical guidelines. |  |  |  |  |  |
| 3. Insufficient policy support or alignment with clinical guidelines affects their effective implementation. |  |  |  |  |  |
| 4. The availability of continuing education opportunities specifically addressing CNS infections impacts Laboratory technologists and microbiologists' adherence to clinical guidelines. |  |  |  |  |  |
| 5. The availability of concise summaries or quick-reference guides for clinical guidelines affects their practical use. |  |  |  |  |  |
| 6. Economic considerations, such as cost implications affect health workers’ practice. |  |  |  |  |  |
| 7. The integration of clinical guidelines into electronic health records impacts their practical utility. |  |  |  |  |  |
| 8. Psychosocial factors, such as stress or burnout, impact health workers' ability to follow clinical guidelines |  |  |  |  |  |
| 9. The accessibility of timely updates and revisions to clinical guidelines impacts their relevance and applicability. |  |  |  |  |  |
| 10. The influence of colleagues in following clinical guidelines affects individual adherence. |  |  |  |  |  |
| 11. Language proficiency, including understanding guidelines/SOPs written in a non-native language, may affect their implementation. |  |  |  |  |  |
| 12. The availability of SOPs facilitates guideline adherence. |  |  |  |  |  |
| 13.  The presence of multidisciplinary healthcare teams positively affects the integration of clinical guidelines into practice. |  |  |  |  |  |

Do you have any additional feedback or comments that you would like to share that are not covered in the questionnaire? If so, please share them with us

……………………………………………………………………………………………………………………………………

Would you be willing to be contacted in the future about this study? If yes, please provide contact info below. If not, skip this question and submit your responses.

…………………………………………………………………………………………………………………………………

**Pharmacist Survey**

**Questionnaire on ‘’Assessment of Adherence to Clinical Guideline Use in the Prevention, Diagnosis, and Treatment of CNS Cryptococcus Infections According to WHO 2022 Guidelines, among Physicians and other Healthcare Providers in Ethiopia’’**

I kindly request your participation in a survey entitled "’ Assessment of Adherence to Clinical Guideline Use in the Prevention, Diagnosis, and Treatment of CNS Cryptococcus Infections According to WHO 2022 Guidelines, among Physicians and other Healthcare Providers in Ethiopia’’. Your expertise and insights are of great value in our survey.

The purpose of this survey is to identify the barriers that healthcare providers face to the effective implementation of clinical guidelines in the treatment of CNS Cryptococcus infections. By understanding these challenges, we aim to improve the quality of care and outcomes for patients with this condition. Your contribution to this study will greatly assist us in capturing a comprehensive perspective on the current state of guideline adherence in Africa ultimately leading to the development of targeted interventions and strategies to improve clinical practice in the region.

The survey questionnaire has been designed to gather information about your experiences, perceptions, and specific challenges faced in practicing guideline recommendations in the context of CNS Cryptococcus infections. Your responses will remain completely anonymous and confidential, ensuring that your individual views are protected. The survey will take approximately 10-15 minutes to complete.

We kindly ask that you submit your responses within two weeks of being sent this survey for data analysis. All responses will be accepted if submitted later.

On behalf of the research team, I would like to express our sincerest gratitude for considering our request to participate in this important study. Your expertise and feedback are invaluable to us, and we highly value the time and effort you will dedicate to completing the survey.

Should you require any further information or have any questions about the study, please do not hesitate to contact me at [mamoblen83@gmail.com](mailto:mamoblen83@gmail.com)

**Section I**

**Demographic Data**

1. What is your title or position within the Pharmacology unit?

…………………………………………………………………………………………………………………..

1. How many years of experience do you have in working with patients who may have CNS Cryptococcal infection?  ( Infectious Disease Pharmacology Division )
   - <1 year
   - 1-5 years
   - 6-10 years
   - >10 years
2. Please indicate the type of your primary practice:
   - Academic Institution
   - Referral hospital
   - District hospital
   - Private practice
3. Where is your primary practice located?
   - Metropolis (population >1,000,000)
   - City (population >100,000 to 1,000,000)
   - Large town (population >50,000 to 100,000)
   - Medium town (population >20,000 to 50,000)
   - Small town (population 2000 to 20,000)
4. Please mention the name of your center:

…………………………………………………………………………………………………………………………….

1. Which guideline informs your approach to cryptococcal disease prevention and management? (Hospital/regional/national/WHO guidelines )…………………………………………………………………………………………………………………………….
2. Please attach pdf document or the link of the guidelines  referenced above if accessible (Hospital/Regional/National/WHO guidelines you use in your facility if you have access)

…………………………………………………………………………………………………………………………….

1. Are you aware of the 2022 WHO Cryptococcal prevention, diagnosis, and treatment guidelines?
   - Yes
   - No
2. Have you received training on the 2022 WHO cryptococcal infection diagnosis guidelines?
   - Yes
   - No
3. If your answer is yes to the above question, What was the date of your training?

--/--/----

1. How easily can you access updated international Cryptococcus treatment guidelines?
   - Very Easily
   - Easily
   - Neither Easy nor difficult
   - Difficult
   - Very Difficult
2. Do you believe there is a need for additional training related to cryptococcal disease diagnosis for Microbiologists?
   - Yes
   - No

**Section II**

**EQUAL Cryptococcus Score 2018**

A simple tool to summarize guideline recommendations that could be used to evaluate guideline adherence as a marker of quality of care and to support antimicrobial stewardship. Please select the most accurate option.

How often are these medications for cryptococcal meningitis treatment available in your facility?

|  | Always | Sometimes | Occasionally | Rarely | Never |
| --- | --- | --- | --- | --- | --- |
| 1. Liposomal Amphotericin B |  |  |  |  |  |
| 2. Amphotericin B deoxycholate |  |  |  |  |  |
| 3. Flucytosine |  |  |  |  |  |
| 4. Fluconazole |  |  |  |  |  |
| 5. HAART |  |  |  |  |  |
| 6. Corticosteroids |  |  |  |  |  |

7. Considering physician practices at your facility, which antifungal medication is most commonly prescribed for treating cryptococcal infections?

- Liposomal Amphotericin B
- Amphotericin B deoxycholate
- Flucytosine
- Fluconazole
- Corticosteroids

8. What factors limit the use of liposomal amphotericin B at your facility?

- Limited availability of the medication
- High cost, making it unaffordable for most patients
- Low demand from physicians for this treatment
- Current treatment guidelines do not recommend Liposomal Amphotericin B
- Other reasons not listed
- Liposomal amphotericin B use is not limited at my facility

If you selected "Other reasons not listed" for #8, please specify below:…………………………………………………………………………………………………………..

 9. What factors limit the use of Amphotericin B deoxycholate at your facility?

- Limited availability of the medication
- High cost, making it unaffordable for most patients
- Low demand from physicians for this treatment
- Current treatment guidelines do not recommend Amphotericin B deoxycholate
- Other reasons not listed
- Amphotericin B deoxycholate use is not limited at my facility

If you selected "Other reasons not listed" for #9, please specify below:…………………………………………………………………………………………………………..

10. What factors limit the use of Flucytosine at your facility?

- Limited availability of the medication
- High cost, making it unaffordable for most patients
- Low demand from physicians for this treatment
- Current treatment guidelines do not recommend flucytosine
- Other reasons not listed
- Flucytosine use is not limited at my facility

If you selected "Other reasons not listed" for #10, please specify below:…………………………………………………………………………………………………………..

11. What factors limit the use of Fluconazole at your facility?

- Limited availability of the medication
- High cost, making it unaffordable for most patients
- Low demand from physicians for this treatment
- Current treatment guidelines do not recommend flucytosine
- Other reasons not listed
- Fluconazole use is not limited at my facility

If you selected "Other reasons not listed" for #11, please specify below:…………………………………………………………………………………………………………..

**Section III**

Assessment of barriers to guideline adherence: Please rate the following statements on a scale from "**Strongly Disagree**" to "**Strongly Agree**."

To what extent do you agree with the following statements regarding barriers to following guidelines for cryptococcal meningitis diagnosis?

|  | Strongly Agree | Agree | Neutral | Disagree | Strongly Disagree |
| --- | --- | --- | --- | --- | --- |
| 1. The clinical guidelines for the treatment of CNS Cryptococcus infections are not well-known among pharmacists. |  |  |  |  |  |
| 2. The accessibility of timely updates and revisions to clinical guidelines impacts their relevance and applicability. |  |  |  |  |  |
| 3. Insufficient policy support or alignment with clinical guidelines affects their effective implementation. |  |  |  |  |  |
| 4. Inadequate training programs on clinical guidelines contribute to their underutilization |  |  |  |  |  |
| 5. Psychosocial factors, such as stress or burnout, impact pharmacists' ability to follow clinical guidelines |  |  |  |  |  |
| 6. Challenges in the daily workflow of pharmacists make it difficult to incorporate clinical guidelines into practice |  |  |  |  |  |
| 7. Economic considerations, such as cost implications affect pharmacists' practice. |  |  |  |  |  |
| 8. The integration of clinical guidelines into electronic health records impacts their practical utility. |  |  |  |  |  |
| 9. The absence of effective feedback mechanisms on guideline adherence prevents continuous improvement. |  |  |  |  |  |
| 10. Understanding guidelines written in a non-native language may affect their implementation. |  |  |  |  |  |
| 11. The availability of decision support tools, such as algorithms, decision trees, concise summaries, or quick-reference guides facilitates guideline adherence. |  |  |  |  |  |
| 12. The presence of multidisciplinary healthcare teams positively affects adherence to guidelines |  |  |  |  |  |
| 13. The influence of colleagues or peer physicians in following clinical guidelines affects individual adherence. |  |  |  |  |  |
| 14. Pharmacists' perception of the efficacy and effectiveness of clinical guidelines influences their willingness to adhere. |  |  |  |  |  |
| 15. The level of public health awareness of CNS Cryptococcus infections influences guideline adherence. |  |  |  |  |  |

Do you have any additional feedback or comments that you would like to share that are not covered in the questionnaire? If so, please share them with us

……………………………………………………………………………………………………………………………………

Would you be willing to be contacted in the future about this study? If yes, please provide contact info below. If not, skip this question and submit your responses.

……………………………………………………………………………………………………………………………………
